# Addition of an oligoglutamate domain to bone morphogenic protein 2 confers binding to hydroxyapatite materials and induces osteoblastic signaling

**DOI:** 10.1371/journal.pone.0217766

**Published:** 2019-05-31

**Authors:** Andrew S. Curry, David T. McPherson, Abby M. Barlow, Nicholas W. Pensa, Michael S. Reddy, Susan L. Bellis

**Affiliations:** 1 Department of Biomedical Engineering, University of Alabama at Birmingham, United States of America; 2 Department of Cell, Developmental, and Integrative Biology, University of Alabama at Birmingham, United States of America; 3 School of Dentistry, University of California, San Francisco, United States of America; Universite de Technologie de Compiegne, FRANCE

## Abstract

Nonautologous bone grafts have limited osteoinductive potential and thus there is substantial interest in reconstituting these graft materials with osteogenic factors such as bone morphogenic protein 2 (BMP2). However, one limitation of this approach is that BMP2 is typically weakly bound to the graft, which can lead to side effects associated with BMP2 dissemination. In the current study we added a hydroxyapatite (HA)-binding domain onto BMP2 to increase coupling to the graft surface. A sequence consisting of eight glutamate residues (E8) was inserted into the C-terminus of BMP2, and the recombinant protein (rBMP2-E8) was expressed in *E*. *coli*. Compared with rBMP2, rBMP2-E8 displayed markedly enhanced binding to HA disks and was better retained on the disks following exposure to vigorous wash steps. Furthermore, rBMP2-E8 was purified using a heparin column, and evaluated for its capacity to stimulate osteoblastic cell signaling. Treatment of SAOS2 cells with rBMP2-E8 induced SMAD 1/5 activation, confirming that the protein retains activity. Collectively these results suggest that the E8 domain serves as an effective tool for improving rBMP2 coupling to graft materials. The increased retention of rBMP2-E8 on the graft surface is expected to prolong BMP2’s osteoinductive activity within the graft site, while simultaneously reducing off-target effects.

## Introduction

Bone grafting is an increasingly common procedure with over 500,000 grafts performed annually in the US and over 2 million worldwide [1, 2]. Bone grafts are a vital component of bone regenerative therapy in orthopedic medicine, maxillofacial reconstruction, and dental surgery [3]. Currently, autologous bone is the “gold standard” material for bone grafting, as it contains all of the necessary cells, signaling factors, and physical properties to promote osteogenesis [4]. However, autografts are inherently limited in quantity and require an extra surgery to acquire [5, 6]. Hence, alternative graft materials are often used as substitutes for autologous bone [2, 3, 7].

Nonautologous graft materials derive from a variety of sources including allograft and xenograft, however these materials must be sterilized and/or chemically treated to make them safe for human use [3, 7]. These processes often degrade the signaling molecules present in native bone, although the mineral matrix, comprised primarily of hydroxyapatite (HA), remains intact. Alloplast materials composed of synthetic HA or β-tri-calcium phosphate (β-TCP) offer another alternative to autografts, however these substrates have no organic components. Given that allograft, xenograft and alloplast have limited osteoinductive potential, there is intensive interest in developing methods for functionalizing these materials with osteogenic factors [8].

Recombinant Bone Morphogenic Protein 2 (rBMP2) has been utilized as an osteoinductive molecule for bone regeneration since it was first FDA-approved for lumbar spinal fusions in 2004 [9]. rBMP2 is a highly active protein, but due to its short half-life, must be delivered on carriers such as a collagen sponge, xenograft or alloplast [10–13]. However, the binding of rBMP2 to carriers is generally very weak, and therefore most of the protein is released within the first couple days [14, 15]. In consequence, supraphysiologic doses of rBMP2 are usually required to achieve a sustained osteoinductive signal within the graft site. This high concentration of rBMP2, along with dissemination of the protein outside of the graft site, have been linked to many of the clinical side effects associated with rBMP2 including chronic inflammation, edema, and ectopic calcification [16–18]. Thus, there is a great need to develop methods that enable better coupling of rBMP2 to its carrier.

In the present study, we utilized a molecular domain found within native bone proteins to improve the binding of rBMP2 to HA materials. This domain is comprised of repeating glutamate residues which bind to the calcium within HA via the carboxylate groups within glutamate [19]. There is an extensive literature highlighting the affinity of glutamate and aspartate residues for calcium phosphate moieties. Oldberg et al. reported that a cluster of glutamic acid residues within bone sialoprotein, as well as an analogous cluster of aspartates within osteopontin, were responsible for the strong binding of these proteins to HA [20]. The role of the coordinative carboxylate–calcium interaction in directing protein/HA interactions has been confirmed by many other investigators. Goldberg et al. used competitive binding studies to show that peptides composed of either glutamic or aspartic acid were capable of displacing high percentages of both bone sialoprotein and osteopontin from HA [21]. Fujisawa et al. further proposed that acidic amino acid-rich domains constitute an entire class of binding domains used by endogenous bone matrix proteins to anchor onto the face of HA crystals [19]. This same group calculated a dissociation constant of 2.4 μM for the binding of a hexaglutamate peptide to HA, and showed that decreasing the number of glutamates within an oligoglutamate peptide reduced the peptide’s affinity for HA [19].

In view of the strong interaction between oligoglutamate and HA, we and others have added this domain to a variety of biomimetic peptides with the goal of enhancing the anchoring of such peptides to the surface of calcium phosphate graft materials. Oligoglutamate domains have been added to DGEA (a collagen-derived peptide), RGD (integrin-binding peptide), and KRSR and FHRRIKA (proteoglycan-binding peptides), in addition to peptides derived from BMP2 and VEGF [22–31]. In every case, the addition of the oligoglutamate sequence improved peptide binding HA, as well as other calcium phosphate graft materials including β-TCP, two types of commercial allograft, and anorganic bovine bone (ABB) [22, 27, 28, 31–34]. Significantly, the oligoglutamate was highly effective at retaining the peptide on the graft *in vivo*. HA or ABB grafts coated with oligoglutamate-modified mimetic peptides were found to retain the peptides for at least 2 months following graft implantation into rat subcutaneous pouches [31, 33].

Importantly, better peptide/graft coupling has been shown to improve osteoregeneration. Our group synthesized a BMP2-derived mimetic peptide with a heptaglutamate (E7) domain and examined the peptide’s osteoinductive activity in a rat mandibular implant model. ABB particles were coated with either unmodified BMP2-derived peptide (BMP2pep) or E7-modified BMP2 peptide (E7-BMP2pep). As compared with BMP2pep-coated ABB, E7-BMP2pep-coated grafts stimulated significantly more new bone formation, confirming that better anchoring of the peptide to ABB was effective in enhancing tissue regeneration [31]. Furthermore, no side effects were noted with E7-BMP2pep/ABB, in stark contrast to the extensive chronic inflammation observed in rats implanted with rBMP2-coated ABB [31]. However, full-length rBMP2 is expected to have greater inherent osteoinductivity than the short BMP2 mimetic peptide, therefore our goal in the current investigation was to clone an oligoglutamate domain, octaglutamate (E8), into rBMP2 in order to increase protein binding to HA.

## Experimental procedures and materials

### Cloning and rBMP2-E8 vector construction

A clone of the human BMP2 gene encoding the pre-pro-protein was obtained from SINO Biological. All PCR and cloning enzymes were from New England Biolabs. The mature coding sequence, encoding Gln283 to the terminal Arg396, was amplified by PCR using *Pfu* Polymerase and the following primers:

Forward (N-term): 5’-CATATGGGTCAAGCCAAACACAAACAGCGG

Reverse (C-term): 5’-CTCGAGTTATTCCTCTTCTTCTTCCTCTTCTTCGCCACCAGCTGCAC CAGCGCGACACCCACAACCCTCCAC

The 5’ primer added a terminal *Nde*I site, as well as a Gly-encoding codon (GGT) between the ATG start and the codon for Gln283. The 3’ primer included sequence for an Ala-Gly-Ala-Ala-Gly-Gly spacer, followed by eight glutamic acids (E8 domain), the stop codon and a terminal *Xho*I site. The PCR product encoding mature BMP2-E8 was cloned into the pCR-Blunt-II (Life Technologies) cloning vector, and verified by DNA sequencing (S1 Fig). The gene was then sub-cloned into pET-21c (Novagen, EMD Millipore), using the *Nde*I and *Xho*I cloning sites.

### Protein expression in E. coli

The pET-BMP2-E8 plasmid was transformed into BL21(DE3) *E*. *coli* and grown at 37°C in LB broth supplemented with Terrific Broth (LB:TB, 4:1, Life Technologies) and ampicillin to 100 μg/mL. Expression of rBMP2-E8 was induced by the addition of IPTG to 0.5 mM. Cells were harvested at three hours post-induction, and the cell pellet was frozen. The cell pellet was thawed and suspended in sonication buffer (50 mM Tris-HCl pH 7.4, 150 mM NaCl) and lysed by sonic disruption. Insoluble inclusion bodies containing the IPTG-induced rBMP2-E8 were isolated by low-speed centrifugation, and inclusion body pellets were washed by re-suspension in sonication buffer and re-centrifugation three more times. The pellets were then suspended and solubilized in denaturing buffer (6 M guanidine-HCl (GnHCl), 100 mM Tris-HCl pH 8.5, 1 mM EDTA, 100 mM DTT). The solubilized protein was dialyzed against the same buffer with succeeding decreases in the concentration of GnHCl to 3, 2, and 1 M. The protein was then dialyzed into refold buffer (0.5 M GnHCl, 100 mM L-Arginine, 100 mM Tris-HCl pH 8.5, 100 mM NaCl, 5 mM EDTA, 2 mM reduced glutathione (GSH), 1 mM oxidized glutathione (GSSG)) and finally into storage buffer (50 mM Tris-HCl pH 8.5, 250 mM NaCl, 3 mM GSH, 1 mM GSSG, 10% glycerol). Hereafter, the refolded inclusion body preparation is referred to as IBP. At the end of this process, we obtained a yield of 10 ml of IBP solution containing 1.35 mg/ml of rBMP2-E8, as measured by ELISA, from a starting liter culture of *E*. *coli*. Aliquots were collected at each step of the expression and isolation procedures and the various samples were resolved by SDS-PAGE using Nu-PAGE Bis-Tris 4–12% gradient gels in MES buffer (Life Technologies), followed by Coomassie Blue staining of the gels.

### Heparin affinity purification

1 ml heparin columns (HiTrap Heparin HP, GE Healthcare) were used to purify rBMP2-E8 from the IBP. In brief, the column was equilibrated with binding buffer (20mM Tris-HCL, 150mM NaCl, 6M Urea, 5mM DTT, pH 7.5) before adding the inclusion body lysate. The lysate was allowed to bind the column for 15 minutes, and then the column was washed extensively with binding buffer to remove unbound proteins. The rBMP2-E8 protein was then eluted from the column with elution buffer (20mM Tris-HCL, 2M NaCl, 6M Urea, 5mM DTT, pH 7.5) and the eluate concentrated using Amicon Ultra-2 mL Ultracel-10K centrifugal filters. The affinity-purified protein was then diluted 1:20 into a refolding buffer (55mM Tris-HCl, 10.56 mM NaCl, 0.44 mM KCl, 550 mM Guanidine-HCl, 2.2 mM MgCl2, 2.2 mM CaCl2, 550 mM L-arginine, 1 mM DTT) as described in [35]. Buffers were exchanged using Ultra-2 mL Ultracel-10K filters.

### Cell signaling and immunoblotting

SAOS2 cells were obtained from ATCC and maintained in Hyclone DMEM (GE Healthsciences) supplemented with 10% fetal bovine serum (FBS, Atlanta Biologicals) and 1% antibiotic/antimycotics (Invitrogen). Prior to signaling experiments, cells were serum-starved for 24 hours. BMP2 activity was assessed by monitoring SMAD 1/5 phosphorylation in cells treated for 15 minutes with either 100 ng/ml of total protein from the inclusion body preparation (quantified by BCA assay, Thermo Fisher) or 100 ng/ml of pure rBMP2 (Peprotech, cat# 120–02). Following this treatment, cells were lysed using radioimmune precipitation assay (RIPA) buffer (Thermo Fisher) containing protease and phosphatase inhibitors (Sigma). Samples were resolved by SDS-PAGE and then transferred to polyvinylidene difluoride (PVDF) membranes (Millipore). Membranes were incubated in 5% nonfat dry milk dissolved in Tris-buffered saline (TBS) containing 0.1% Tween-20 (TBST). The blots were probed with antibodies for pSMAD 1/5 (Cell Signaling Technology, cat# 9516), SMAD 1 (Cell Signaling Technology, cat# 9743), or rBMP2 (Thermo Fisher, cat# MA1-26766). Subsequently, blots were incubated with the appropriate HRP-conjugated secondary antibodies (Cell Signaling). Blots were also probed with anti-β-tubulin or anti-β actin (both from Abcam) to control for protein loading. Proteins were detected by enhanced chemiluminescence using Clarity Western ECL substrate (BioRad, cat# 170–5060).

### ELISA for BMP2 solutions

Relative levels of BMP2 in the inclusion body preparation vs. commercial rBMP2 solution were evaluated using an enzyme-linked immunosorbent assay (ELISA). An ELISA plate was coated overnight with a rBMP2 capture antibody (Peprotech, cat# 900-T255) and then washed and blocked with 0.1% BSA. Afterwards, varying concentrations of total protein from the inclusion body preparation (measured by BCA), as well as a known reference amount (5 ng/ml) of commercial rBMP2 (Peprotech), were added to the plate and allowed to bind for 2 hours. Plates were subsequently washed and incubated for 2 hours with a distinct rBMP2 antibody used for detection (Thermo Fisher, cat# MA1-26766). A biotinylated secondary antibody was added, followed by streptavidin-HRP. The BMP2 signal was monitored by adding a TMB colorimetric substrate (Thermo Fisher), and then quantifying absorbance (optical density, OD) using a plate reader.

### Modified ELISA for BMP2 bound to HA disks

HA disks were prepared from hydroxyapatite fast flow powder (MP Biomedicals) using a 15.875 mm die under 3000 psi as in our prior publications [22]. Afterwards, the disks were sintered at 1000˚C in a Thermolyne 48000 furnace for 3 hours before being allowed to slowly cool to room temperature. Disks made by this method have previously been found by our group to have a highly crystalline apatite structure as confirmed by x-ray diffraction using a Siemens D500 Diffractometer [36]. Additionally, profilometry of the surface of the disks calculated the arithmetic average roughness (R_A_) to be 0.921 μm using a Federal Surfanalyzer 4000 [36]. Disks were sterilized by autoclaving before use. For the modified ELISA, HA disks were coated with either 100 ng/ml rBMP2 (Peprotech) or 100 ng/ml of total protein from the inclusion body preparation. Disks were coated for either 1 hour at room temperature or overnight at 4˚C. HA disks coated with 1% BSA served as a negative control. After coating, disks were washed briefly with TBS, or alternatively, incubated in TBS for 5 days on a shaker in 4˚C. After the wash steps, disks were blocked in 1% BSA for 30 minutes at room temperature. Disks were washed with TBST and then incubated for 1 hour at room temperature with a rabbit anti-human rBMP2 antibody (Peprotech, cat# 500-P195) at 1 μg/ml in TBS containing 0.1% BSA. Disks were washed again with TBST and incubated with a donkey anti-rabbit Alexa Fluor 488 (Invitrogen, cat# A21206) at 4 μg/ml in TBS/0.1% BSA for 1 hour at room temperature. The disks were washed with TBST and imaged using a fluorescent microscope. ImageJ software was used to quantify the fluorescence signal of each disk.

## Results

### Expression of rBMP2-E8 in E. coli

**Fig 1:**
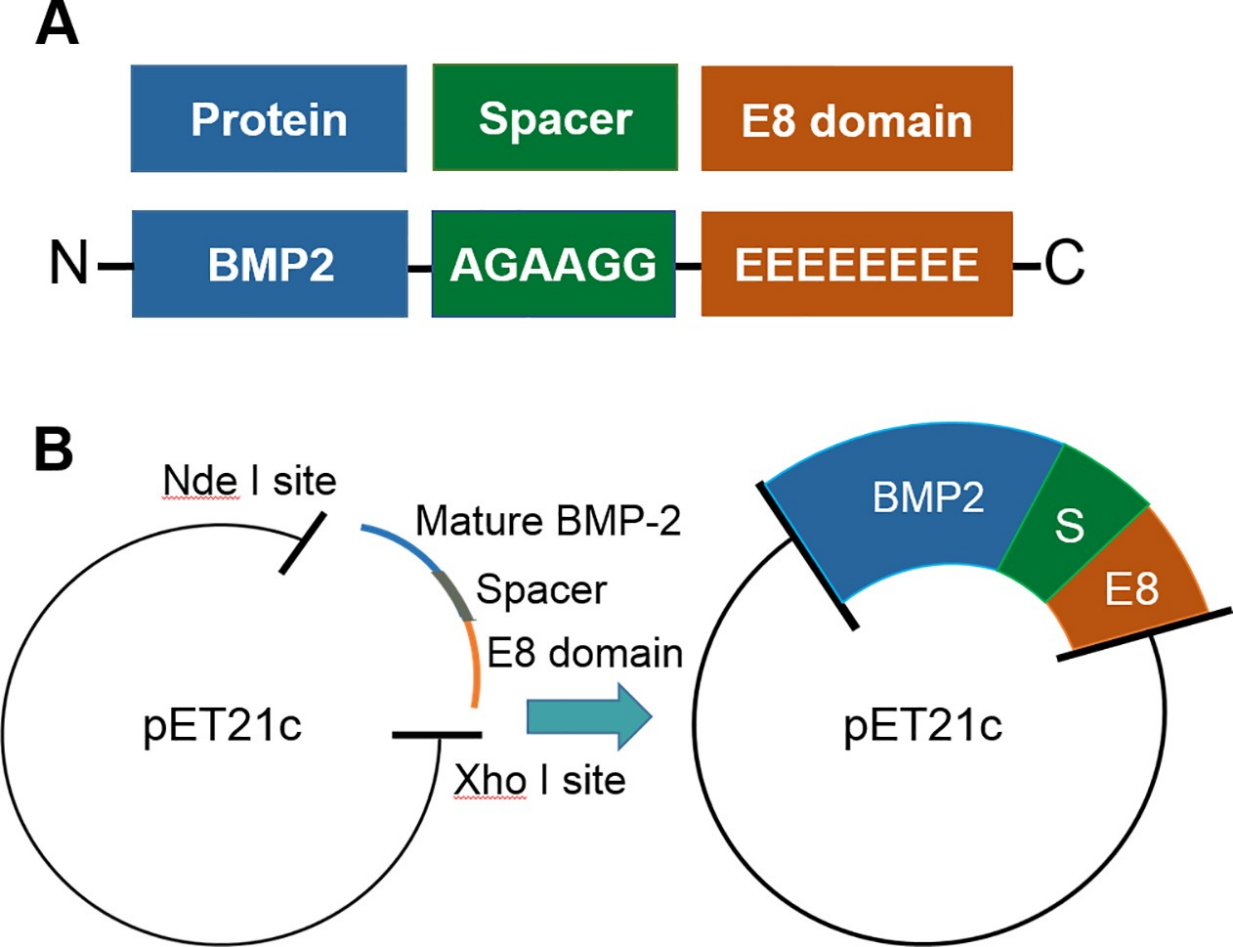
Cloning scheme for rBMP2-E8. (A) PCR cloning was utilized to add the E8 domain to the C-terminus of the mature rBMP2 sequence, along with an intervening spacer domain consisting of alanine and glycine residues. The construct was verified by DNA sequencing. (B) The rBMP2-E8 construct was cloned into the pET21c vector for expression in *E*. *coli*.

The native BMP2 molecule contains a pro-domain that must be enzymatically cleaved before the protein becomes active. We therefore cloned the E8 domain into the DNA sequence encoding the mature BMP2 protein (i.e., lacking the pro-domain). E8 was added to the C-terminus of BMP2, along with an intervening spacer sequence composed of alanine and glycine residues (Fig 1A). This spacer served to separate the negatively-charged E8 domain from the BMP2 sequence. The resulting recombinant BMP2-E8 construct (rBMP2-E8) was sequenced and compared with the human BMP2 reference sequence, which confirmed fidelity of the construct (S1 Fig).

**Fig 2:**
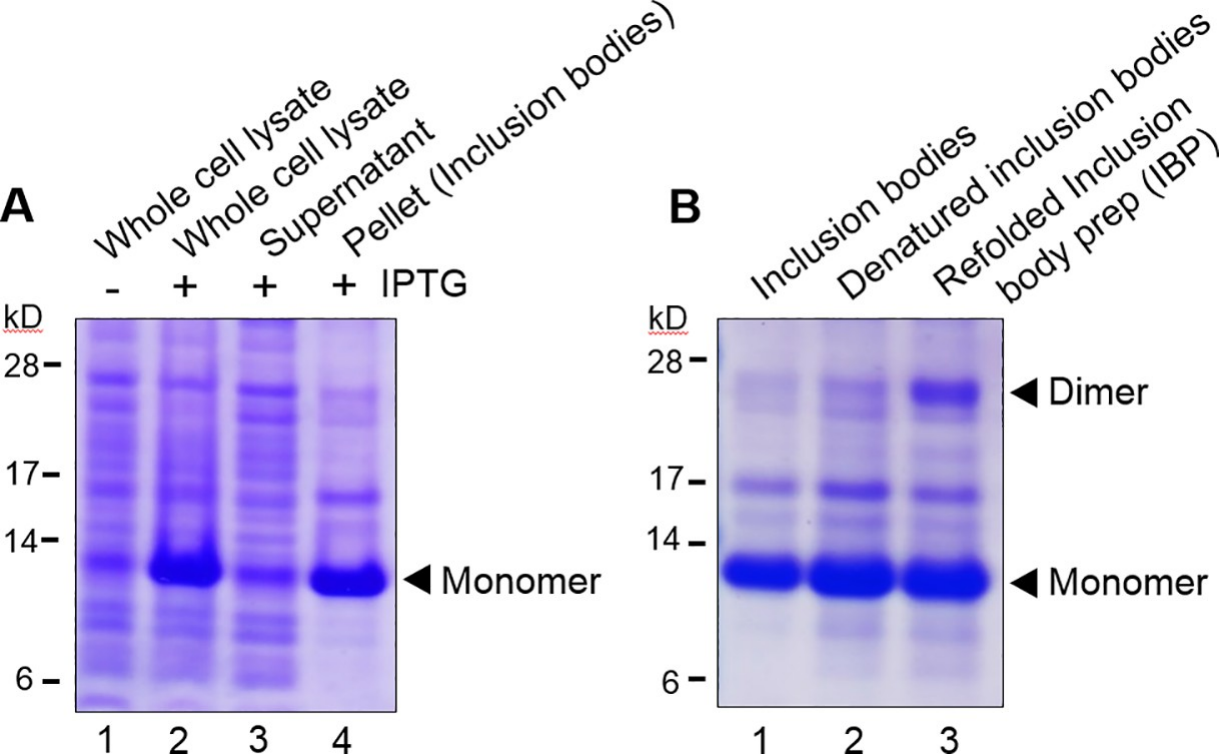
rBMP2-E8 expression in *E*. *coli* localizes primarily to inclusion bodies. (A) Coomassie-stained gels reveal IPTG-induced expression of a ~12 kD protein, a mass consistent with the rBMP2-E8 monomer. Lanes 1 and 2 depict whole cell lysates from cells treated with or without IPTG. Lanes 3 and 4 represent the supernatant (soluble proteins) and pellet (inclusion body fraction), respectively, following centrifugation of lysates from IPTG-treated cells. (B) Dialysis of the GnHCl-solubilized inclusion body protein in refolding buffer enriches for the putative, 26 kD rBMP2-E8 dimer. The insoluble inclusion body protein is shown in lane 1. Lane 2 depicts the denatured inclusion body lysate and lane 3 indicates the refolded inclusion body preparation (IBP).

The rBMP2-E8 construct was then cloned into the pET21c vector between the Nde I and Xho I sites (Fig 1B). *E*. *coli* were transfected with the pET21c/rBMP2-E8 vector, and protein expression was induced by IPTG. Cells were lysed, and then whole cell lysates resolved by SDS-PAGE, followed by Coomassie staining of the gels (Fig 2A). As compared with lysates from uninduced cells (lane 1), lysates from IPTG-treated cells (lane 2), displayed a dramatic increase in the abundance of a ~12 kD protein, a mass consistent with the MW of the BMP2 monomer. We next determined whether the putative 12 kD rBMP2-E8 protein was present in inclusion bodies, given that inclusion body formation is commonly observed during production of rBMP2 in *E*. *coli* [35, 37]. IPTG-treated cells were lysed by sonication and centrifuged to separate soluble proteins (supernatant) from the inclusion bodies (pellet). As shown in Fig 2A, the great majority of the 12 kD rBMP2-E8 monomer was found in the pelleted fraction (compare lanes 3 and 4), suggesting that the protein localized primarily to inclusion bodies.

The active form of native BMP2 is a dimer held together by a strong di-sulfide bond. Results in Fig 2A suggested that rBMP2-E8 was primarily expressed in the inactive monomeric form. Accordingly, we adopted a number of steps to renature the protein (Fig 2B). First, proteins within the inclusion body lysate were solubilized using a GnHCl denaturing buffer to disrupt the molecular interactions (lane 2). Subsequently, the preparation was dialyzed into a refolding buffer (lane 3) to facilitate formation of a native conformation, including the formation of the critical di-sulfide bond that drives BMP2 dimerization. Following incubation in the refolding buffer, a substantial increase in a ~26 kD protein was noted, consistent with the formation of a rBMP2-E8 dimer. However, while the refolding step restored some degree of rBMP2-E8 dimerization, a significant amount of the monomeric rBMP2-E8 remained in the refolded fraction.

### The E8 domain mediates rBMP-E8 binding to HA

**Fig 3:**
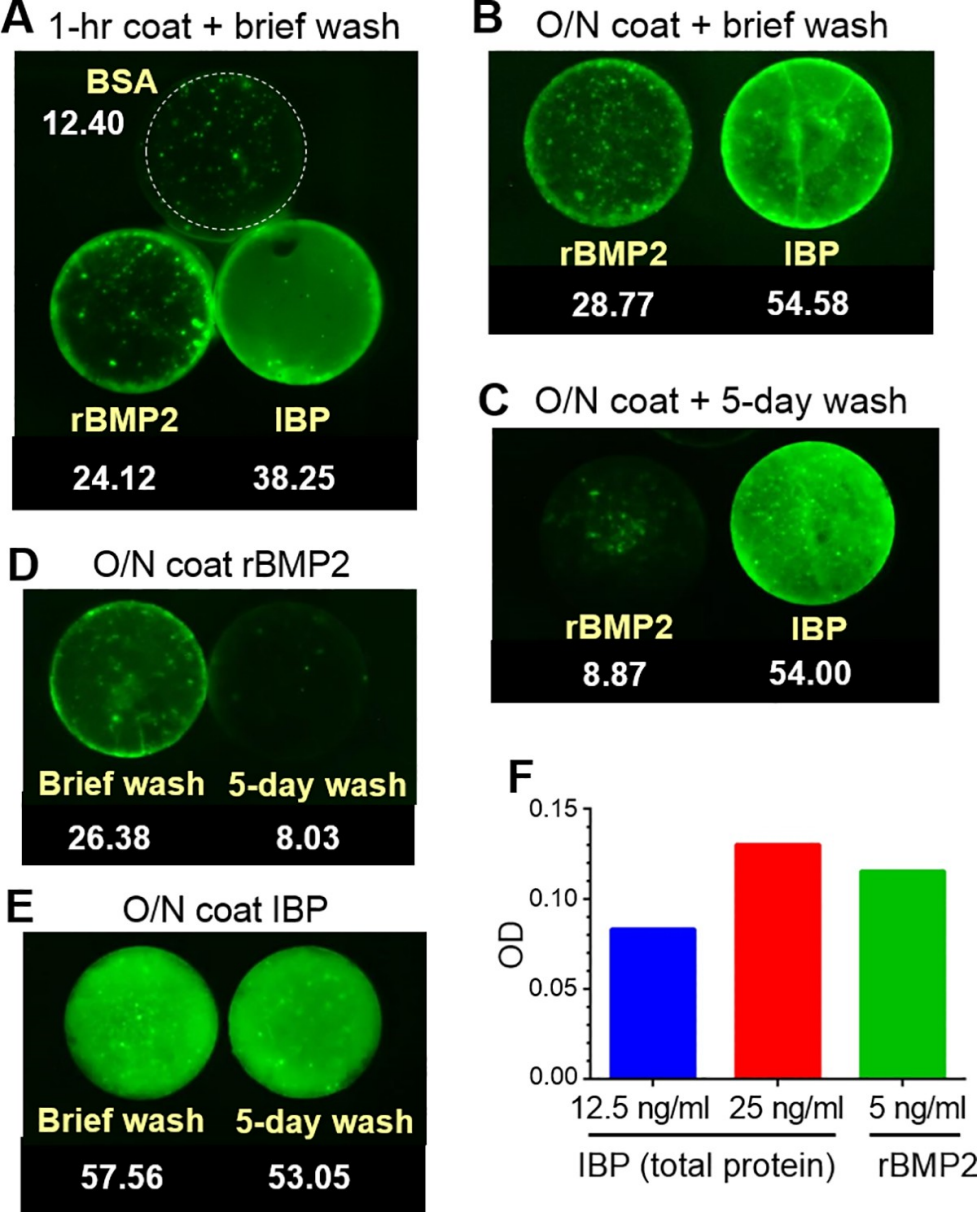
E8 domain confers anchoring of rBMP2-E8 to HA disks. (A) HA disks were coated for 1 hour with BSA, 100 ng/ml commercial rBMP2, or 100 ng/ml total protein of the refolded inclusion body preparation (IBP). Disks were washed briefly with TBS and a modified ELISA was used to detect bound BMP2. For the modified ELISA, disks were incubated with an anti-rBMP2 antibody, followed by a fluorescent secondary antibody, and then disks were imaged using fluorescence microscopy. (B &C) Disks were coated overnight (O/N) with rBMP2 or IBP in 4°C and then washed briefly (B) or washed on a shaker for 5 days (C), prior to imaging. (D&E) Side-by-side comparisons of brief, vs. 5-day, washes of disks coated overnight with either rBMP2 (D) or IBP (E). ImageJ was used to quantify the amount of signal on each disk and these measurements are displayed under each disk label. (F) The concentration of BMP2 in the IBP was estimated using a BMP2 ELISA. 12.5 and 25 ng/ml of total IBP protein (quantified by a BCA assay) was compared with a known concentration (5 ng/ml) of pure rBMP2.

Having generated the rBMP2-E8 protein, we next examined whether the E8 domain was effective in directing protein binding to HA materials. While oligoglutamate domains have been previously used to anchor short synthetic peptides onto HA, this approach has not been utilized with full-length proteins, which have considerably greater mass. To evaluate protein binding, HA disks were coated with equivalent concentrations of either a commercial source of rBMP2, or the refolded rBMP-E8-containing inclusion body preparation (hereafter referred to as “IBP”). The protein concentration of the IBP was quantified by BCA assay. Disks were also coated with BSA as a negative control. After a 1-hour coating with BSA, rBMP2, or IBP, disks were washed briefly to remove unbound proteins, and a modified ELISA assay for BMP2 was used to detect proteins bound to the disks. Specifically, coated disks were incubated with an anti-BMP2 antibody, followed by a fluorescent secondary antibody. Disks were washed in TBS, and then imaged by fluorescence microscopy (disks were placed within the same microscopic field to allow a direct comparison). As shown in Fig 3A, an increase in fluorescence signal was detected on disks coated with IBP as compared with commercial rBMP2, suggesting that a greater amount of rBMP-E8 was bound to HA relative to rBMP2. As expected, no detectable fluorescence was observed on BSA-coated disks, confirming specificity of the assay for BMP2. In a second set of experiments, HA disks were coated overnight with rBMP2 or IBP, and similar to results in Fig 3A, enriched binding of rBMP-E8 to HA was observed in comparison with rBMP2 (Fig 3B). These data suggest that the increased binding of rBMP2-E8 to HA allows more BMP2 protein to be anchored onto HA materials.

To test the strength of the interaction between rBMP2-E8 and HA, we coated HA disks overnight with IBP or rBMP2, and then washed the disks at 4˚C with agitation for 5 days. At the end of this interval, the difference in fluorescence signal between disks coated with rBMP2 or IBP was even more striking (Fig 3C). The more thorough washing of disks over 5 days removed most of the initially-bound rBMP2, while rBMP2-E8 stayed securely anchored. As shown in a side-by-side comparison of rBMP2-coated disks treated with the brief vs. 5-day wash (Fig 3D), there was a drastic reduction in bound rBMP2 over the 5-day wash period. Contrarily, no apparent loss in bound rBMP2-E8 was observed over this same interval (Fig 3E). Thus, the E8/HA bond was strong enough to withstand stringent wash conditions.

Notably, the amount of rBMP2 in the initial coating solutions was actually in excess of that of rBMP2-E8. For the modified ELISA, 100 ng/ml of either total IBP protein (measured by BCA) or commercial rBMP2 (pure rBMP2) was used to coat disks, however the IBP clearly contains many proteins other than rBMP2-E8. To estimate the amount of rBMP2-E8 within the IBP, we used an ELISA for BMP2. Total IBP protein was added to the ELISA in either 12.5 or 25 ng/ml concentrations. As a reference, 5 ng/ml of commercial rBMP2 was also included. These studies indicated that ~20 ng/ml of total IBP protein had the equivalent of 5 ng/ml of rBMP2, suggesting that ~25% of the total protein within IBP was comprised of rBMP2-E8. In view of these data, the binding differences shown in Fig 3 are even more compelling, since the concentration of rBMP2 in coating solutions was approximately 4 times that of rBMP-E8.

### Inclusion body preparation fails to activate BMP2 signaling

**Fig 4:**
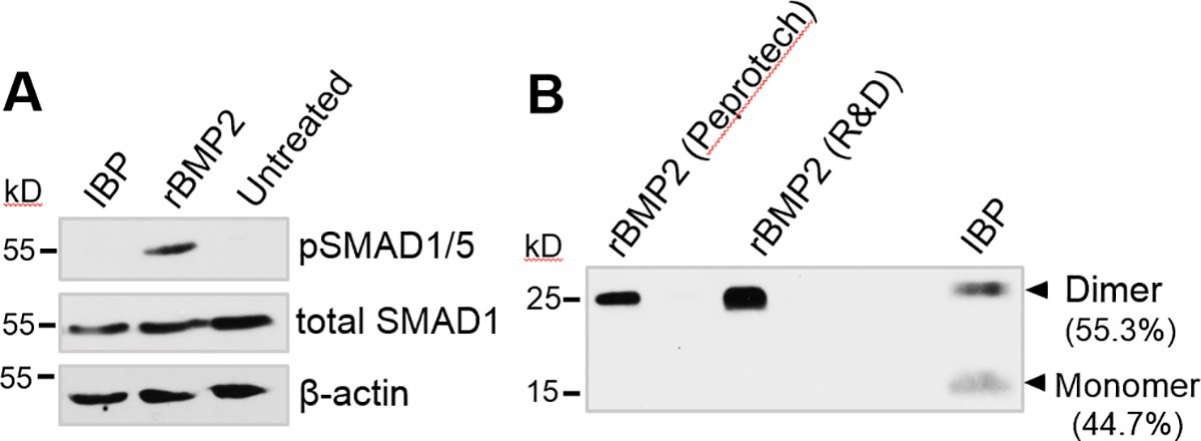
Inclusion body prep fails to activate BMP2 signaling in osteoblastic cells. (A) SAOS2 osteoblastic cells were treated for 15 minutes with the IBP or rBMP2, or left untreated, and then cells were lysed and monitored for phosphorylated SMAD 1/5 by immunoblotting. Strong activation of pSMAD 1/5 was elicited by rBMP2, but not IBP. (B) Immunoblots of BMP2 from IBP or rBMP2 from two different commercial sources (Peprotech and R&D Systems). ImageJ was used to quantify the percentage of rBMP2-E8 in either the dimer or monomer form. This data is represented under each HA disk label. Both the BMP2 monomer and dimer were present in the IBP, whereas only the dimerized BMP2 isoform was found in commercial rBMP2.

Having confirmed that the E8 domain confers binding for HA, we next evaluated whether the rBMP2-E8-containing IBP was capable of activating BMP2-dependent signaling. SAOS2 osteoblastic cells were treated for 15 minutes with IBP, rBMP2, or left untreated, and then cells were lysed to probe for pSMAD 1/5, a marker of BMP2 signaling. As shown in Fig 4A, rBMP2 stimulated substantial phosphorylation (activation) of SMAD 1/5, whereas IBP had no effect. There are a number of factors that may account for the lack of activity by the IBP fraction. First, IBP contains proteins other than rBMP2-E8, and it is possible that some *E*. *coli*-derived proteins may interfere with cell signaling. Secondly, data shown in Fig 2B indicated that most of the rBMP2-E8 within the IBP was present in the monomeric form, which is inactive. Furthermore, the monomeric form may actually interfere with signaling by the rBMP2 dimer by competing for surface BMP receptors, as has been reported for native BMPs [38]. To further assess relative levels of BMP2 monomers and dimers, we conducted Western blots for BMP2. The IBP proteins, along with two different commercial rBMP2 proteins, were resolved on SDS-PAGE gels, transferred to PVDF membranes, and immunoblotted with an anti-BMP2 antibody. As shown in Fig 4B, both of the commercial rBMP2s were present entirely in the dimeric, active form. In contrast, a significant amount of monomer was noted in the IBP lysate. Because of the potential inhibitory effects of either the monomer, or other *E*. *coli*-derived proteins, on cell signaling, we proceeded to purify rBMP2-E8 from the IBP.

### Purification of rBMP2-E8

**Fig 5:**
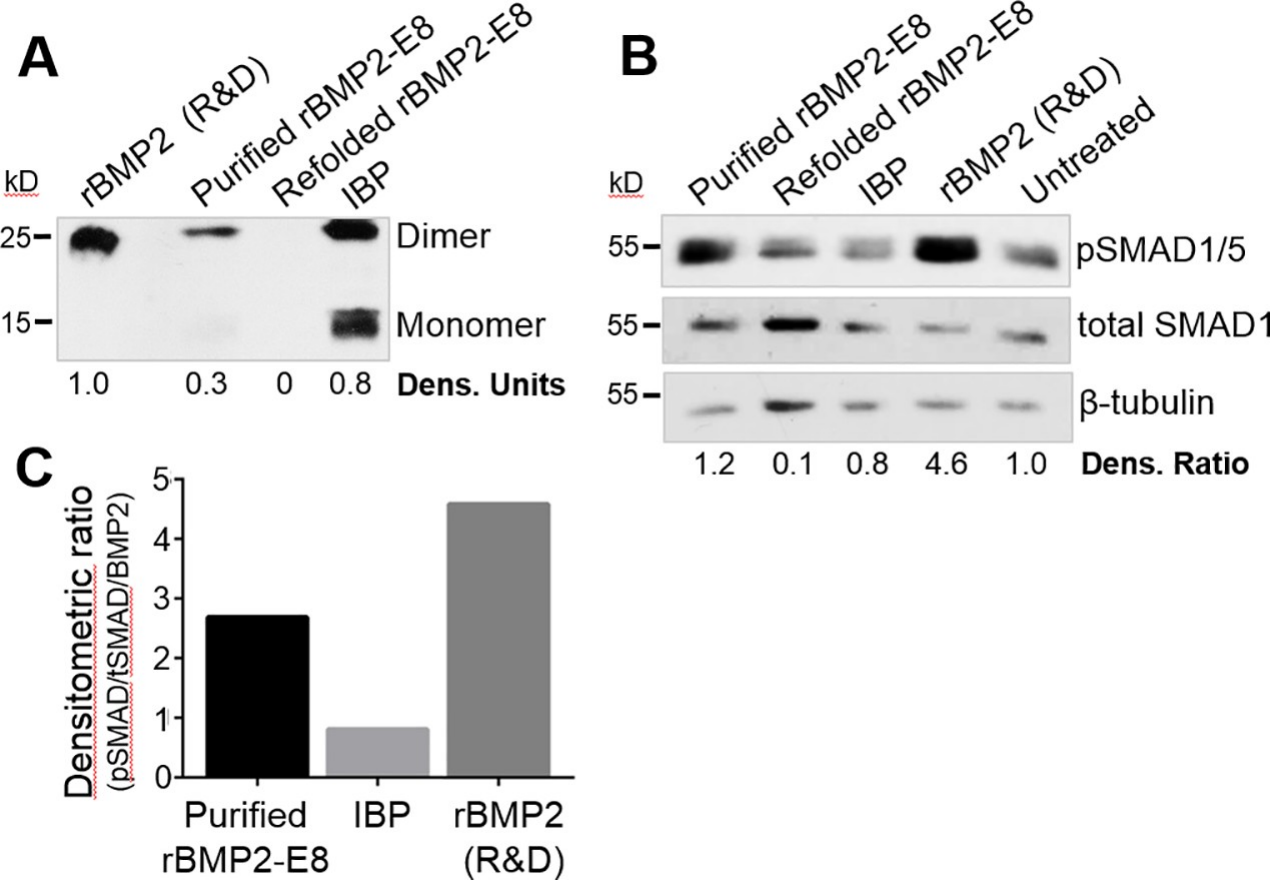
Affinity-purified rBMP2-E8 is active in stimulating osteoblastic signaling. (A) rBMP2-E8 was purified using a heparin affinity column (referred to as purified rBMP2-E8), or for some experiments, the purified protein was further resuspended in a refolding buffer (“refolded rBMP2-E8”). BMP2 immunoblots were conducted for purified rBMP2-E8, refolded rBMP2-E8, IBP, and commercial rBMP2. (B) SAOS2 cells were either left untreated or treated for 15 minutes with purified rBMP2-E8, refolded rBMP2-E8, IBP, or rBMP2. Cell lysates were then immunoblotted for SMAD 1/5 activation. Densitometry was used to compare levels of pSMAD 1/5 to total SMAD 1, and then this ratio was normalized to the β-tubulin loading control. Densitometric ratios are shown below the blots. (C) Densitometric ratios for pSMAD 1/5/total SMAD 1, as depicted in panel B, were normalized to the amount of BMP2 present in the various solutions (estimated from densitometry of BMP2 blots shown in panel A).

Heparin affinity columns are commonly used to purify rBMP2 from *E*. *coli*, we therefore adopted this approach. Using standard protocols [35], heparin-sepharose columns were equilibrated with binding buffer, and then loaded with IBP lysate. IBP proteins were allowed to bind the column for 15 minutes, and then unbound proteins were removed from the column using 5 washes of binding buffer. The bound rBMP2-E8 was subsequently eluted from the column using a high salt elution buffer. We also attempted to refold the protein by replacing the elution buffer with a refolding buffer. Immunoblotting was then conducted to assess BMP2 dimerization state in samples collected immediately after elution (abbreviated as “purified rBMP2-E8”), as well as samples that were suspended in refolding buffer (“refolded rBMP-E8”). These samples were compared with the original IBP and commercial rBMP2 (Fig 5A). In contrast to the presence of both monomer and dimer in the IBP lysate, the eluted rBMP2-E8 appeared to contain only the dimerized form, suggesting that rBMP2-E8 dimers may have preferentially bound to the heparin column, or alternatively, the affinity purification process may have aided in dimer formation. Once the dimer is formed, it is expected to be very stable. Dimerization of BMPs, as well as other members of the TGF-β family, is mediated by a single, well-conserved di-sulfide bond. This bond lies within a protected cysteine knot, which makes the di-sulfide linkage highly resistant to reducing agents [39]. In contrast to the purified rBMP2-E8 sample, the refolded rBMP2-E8 fraction unexpectedly lacked detectable protein by Western blot. These data suggest that the protein was lost somewhere in the refolding process, perhaps due to protein aggregation.

### Purified rBMP2-E8 stimulates BMP2 signaling

Having used affinity chromatography to remove *E*. *coli* proteins and enrich for the rBMP2-E8 dimer, we next tested the activity of the purified rBMP2-E8. SAOS2 cells were either left untreated or incubated with purified rBMP2-E8, the refolded rBMP2-E8 sample, IBP, or commercial rBMP2. As before, cells were lysed and probed for pSMAD 1/5. Significantly, purified rBMP2-E8 induced a marked increase in SMAD 1/5 phosphorylation relative to the untreated control, confirming activity (Fig 5B). Contrarily, neither IBP nor the refolded sample (which, in fact, lacked rBMP2-E8) induced any activation of pSMAD 1/5. To quantify activity, densitometric analyses were conducted to compare pSMAD with total SMAD levels, and this ratio was then normalized to the β-tubulin loading controls.

While purified rBMP2-E8 was clearly able to stimulate osteoblastic cell signaling, it is important to note that the actual amount of rBMP2-E8 varied within the IBP, the purified rBMP2-E8, and refolded rBMP2-E8 preparations, as seen in Fig 5A. We thus normalized pSMAD 1/5 activation, as measured by the densitometric ratios shown in Fig 5B, to the amount of rBMP2-E8 or rBMP2, which was quantified by densitometric analyses of the BMP2 immunoblots in Fig 5A. As depicted in Fig 5C, when normalized to the amount of rBMP2-E8 or rBMP2, the activity of purified rBMP2-E8 approached that of commercial rBMP2. These results suggest that the addition of an oligoglutamate domain to rBMP2 can be used to significantly improve coupling of active rBMP2 to HA graft materials.

## Discussion

The aim of the present study was to generate a recombinant BMP2 protein with an added E8 domain to improve the anchoring of rBMP2 to bone graft materials. The E8 domain was cloned into the C-terminus of the mature rBMP2, because sequences within the N-terminus are known to regulate protein stability [40] as well as bioactivity [41, 42]. Additionally, other motifs, such as GFP and His tags, have been previously inserted into the BMP2 C-terminus without affecting rBMP2 activity [43]. Given that the E8 domain is considerably smaller than tags like GFP, the addition of E8 to the C-terminus was not expected to significantly alter the conformation or function of rBMP2. The rBMP2-E8 construct was expressed in *E*. *coli*, and as with other studies of rBMP2 production in *E*. *coli* [35, 37], rBMP2-E8 was stored in inclusion bodies after IPTG induction.

The rBMP2-E8 protein was compared to rBMP2 for its capacity to bind HA graft materials. We found that a greater amount of rBMP2-E8 bound to HA disks than rBMP2 during initial coating steps, consistent with the known binding of oligoglutamate domains to HA [19, 22, 23, 27–29, 33, 34]. Furthermore, a pronounced enhancement in rBMP2-E8 retention on HA was observed when disks were exposed to vigorous wash steps. After a 5-day wash with agitation, nearly all of the rBMP2 was removed from HA, whereas no detectable loss of rBMP2-E8 was observed over this same time interval. These results are significant because they show that the E8 domain is highly effective in coupling a full-length protein to HA materials. In prior investigations, oligoglutamate sequences were added to short synthetic peptides, which have drastically smaller mass than rBMP2. Also important, our results infer that, *in vivo*, rBMP-E8-coupled grafts would likely be able to withstand shear forces and other perturbants that would remove passively-adsorbed rBMP2 from the graft surface.

Having confirmed efficient binding of rBMP2-E8 to HA, we evaluated the protein’s capacity to stimulate BMP2-related signaling events such as SMAD 1/5 activation. In initial experiments, SAOS2 osteoblastic cells were treated with the crude rBMP2-E8-containing inclusion body preparation, however this failed to activate SMAD 1/5. The reasons for the lack of activity remain unclear, but may relate to the presence of inhibitory *E*. *coli* proteins within the lysate, improper rBMP2-E8 folding, and/or the large amount of rBMP2-E8 monomer present within the inclusion body preparation. Not only is the rBMP2 monomer inactive, but it has been reported that the monomer can interfere with signaling by the active, rBMP2 dimer by competing for BMP2 receptors [38]. Thus, we proceeded to isolate rBMP-E8 from the inclusion body preparation by purifying the protein on a heparin affinity column. Heparin columns have been widely used to purify rBMP2 by other investigators [35, 37, 39], and rBMP2 is known to have specific affinity for heparin [44–46]. In addition to removing *E*. *coli* host proteins, we found that purification of rBMP2-E8 on the heparin column greatly enriched for the rBMP2-E8 dimer. Most importantly, the affinity-purified rBMP2-E8 protein was able to stimulate robust SMAD 1/5 activation in osteoblastic cells.

The ability to tether rBMP2 to bone graft materials addresses a number of deficiencies associated with the therapeutic use of rBMP2. In the clinic, rBMP2 is typically delivered on a carrier due to instability of the rBMP2 protein. However, because the binding of rBMP2 to most carriers is weak, the bulk of rBMP2 is released within the first few days [14, 15]. For this reason, high doses of rBMP2 are required to ensure that a sufficient amount of rBMP2 remains bound to the carrier after the initial burst release. Another major concern is that the release of rBMP2 into systemic circulation is associated with multiple deleterious side effects including ectopic calcification, inflammation and increased cancer risk [16, 47]. Based on findings reported in the present study, we anticipate that the E8-mediated anchoring of rBMP2 on graft materials would enable greater amounts of rBMP2 to be initially bound onto the graft, while simultaneously prolonging the retention of the protein within the graft site. This would lower the concentration of rBMP2 needed, reducing both the cost of treatment and risks associated with supraphysiologic doses. Furthermore, better coupling of rBMP2 to the graft would reduce rBMP2 dissemination, thereby limiting off-targets, and extending the osteoinductive signal within the graft site.

Another advantage of rBMP2-E8 is that the protein is expected to bind to a wide range of clinical bone graft materials. In the current study we evaluated rBMP2-E8 binding to HA disks, however prior work has established that oligoglutamate domains are very effective in anchoring peptides to a multiplicity of calcium phosphate materials including various forms of allograft, xenograft and alloplast [22, 32–34]. This enhances clinical versatility, since each of these diverse graft materials has unique properties including degradation kinetics, mechanical stability, porosity, etc. In the aggregate, results herein suggest that the E8 domain holds promise for anchoring rBMP2, and potentially other bone-inducing factors, onto a preponderance of bone graft products and materials already on the market and in use by clinicians and researchers.

## Conclusion

This study has shown that a novel recombinant BMP2 protein with an added HA-binding domain, E8, not only anchors tightly onto HA, but also induces osteoblastic signaling. Better retention of rBMP2-E8 on HA grafts, as compared with unmodified rBMP2, is expected to enable sustained delivery of BMP2 activity within the graft site, promoting new bone formation. Furthermore, the enhanced coupling of rBMP2-E8 to HA materials could lower the unpredictability of rBMP2 treatment by limiting side effects and reducing the amount of protein needed.

## Supporting information

S1 Fig DNA sequencing of rBMP2-E8 vector construct. (TIF)

S2 Fig Original Coomassie stained gel of rBMP2-E8 expression in *E. coli*. (TIF)

S3 Fig Original western blot images from IBP treatment in solution. (TIF)

S4 Fig Original western blot image comparing the IBP to commercial rBMP2. (TIF)

S5 Fig Original western blot image comparing purified rBMP2-E8 to commercial rBMP2 and the IBP. (TIF)

S6 Fig Original western blot images of purified rBMP2-E8 treatment in solution. (TIF)

## References

[1] Campana V, Milano G, Pagano E, Barba M, Cicione C, Salonna G, et al Bone substitutes in orthopaedic surgery: from basic science to clinical practice. J Mater Sci Mater Med. 2014;25(10):2445–61. 10.1007/s10856-014-5240-2 24865980 PMC4169585

[2] Giannoudis PV, Dinopoulos H, Tsiridis E. Bone substitutes: an update. Injury. 2005;36 Suppl 3:S20–7. 16188545 10.1016/j.injury.2005.07.029

[3] Salem D, Natto Z, Elangovan S, Karimbux N. Usage of bone replacement grafts in periodontics and oral implantology and their current levels of clinical evidence—A systematic assessment. J Periodontol. 2016;87(8):872–9. 10.1902/jop.2016.150512 27058348

[4] Marino JT, Ziran BH. Use of solid and cancellous autologous bone graft for fractures and nonunions. Orthop Clin North Am. 2010;41(1):15–26. 10.1016/j.ocl.2009.08.003 19931049

[5] Banwart JC, Asher MA, Hassanein RS. Iliac crest bone graft harvest donor site morbidity. A statistical evaluation. Spine. 1995;20(9):1055–60. 7631235 10.1097/00007632-199505000-00012

[6] Fernyhough JC, Schimandle JJ, Weigel MC, Edwards CC, Levine AM. Chronic donor site pain complicating bone graft harvesting from the posterior iliac crest for spinal fusion. Spine. 1992;17(12):1474–80. 1471005 10.1097/00007632-199212000-00006

[7] Bhatt RA, Rozental TD. Bone graft substitutes. Hand Clin. 2012;28(4):457–68. 10.1016/j.hcl.2012.08.001 23101596

[8] Fisher DM, Wong JM, Crowley C, Khan WS. Preclinical and clinical studies on the use of growth factors for bone repair: a systematic review. Curr Stem Cell Res Ther. 2013;8(3):260–8. 23317434 10.2174/1574888x11308030011

[9] Gautschi OP, Frey SP, Zellweger R. Bone morphogenetic proteins in clinical applications. ANZ J Surg. 2007;77(8):626–31. 10.1111/j.1445-2197.2007.04175.x 17635273

[10] Grabowski G, Cornett CA. Bone graft and bone graft substitutes in spine surgery: current concepts and controversies. J Am Acad Orthop Surg. 2013;21(1):51–60. 10.5435/JAAOS-21-01-51 23281471

[11] Tannoury CA, An HS. Complications with the use of bone morphogenetic protein 2 (BMP-2) in spine surgery. Spine J. 2014;14(3):552–9. 10.1016/j.spinee.2013.08.060 24412416

[12] Kim HS, Park JC, Yun PY, Kim YK. Evaluation of bone healing using rhBMP-2 soaked hydroxyapatite in ridge augmentation: a prospective observational study. Maxillofac Plast Reconstr Surg. 2017;39(1):40 10.1186/s40902-017-0138-9 29302589 PMC5742315

[13] Boden SD, Kang J, Sandhu H, Heller JG. Use of recombinant human bone morphogenetic protein-2 to achieve posterolateral lumbar spine fusion in humans: a prospective, randomized clinical pilot trial: 2002 Volvo Award in clinical studies. Spine. 2002;27(23):2662–73. 10.1097/01.BRS.0000035320.82533.06 12461392

[14] Liu Y, Huse RO, de Groot K, Buser D, Hunziker EB. Delivery mode and efficacy of BMP-2 in association with implants. J Dent Res. 2007;86(1):84–9. 10.1177/154405910708600114 17189469

[15] Boden SD, Martin GJ Jr., Morone MA, Ugbo JL, Moskovitz PA. Posterolateral lumbar intertransverse process spine arthrodesis with recombinant human bone morphogenetic protein 2/hydroxyapatite-tricalcium phosphate after laminectomy in the nonhuman primate. Spine. 1999;24(12):1179–85. 10382242 10.1097/00007632-199906150-00002

[16] James AW, LaChaud G, Shen J, Asatrian G, Nguyen V, Zhang X, et al A Review of the Clinical Side Effects of Bone Morphogenetic Protein-2. Tissue Eng Part B Rev. 2016;22(4):284–97. 10.1089/ten.TEB.2015.0357 26857241 PMC4964756

[17] Haidar ZS, Hamdy RC, Tabrizian M. Delivery of recombinant bone morphogenetic proteins for bone regeneration and repair. Part A: Current challenges in BMP delivery. Biotechnol Lett. 2009;31(12):1817–24. 10.1007/s10529-009-0099-x 19690804

[18] Shimer AL, Oner FC, Vaccaro AR. Spinal reconstruction and bone morphogenetic proteins: open questions. Injury. 2009;40 Suppl 3:S32–8. 20082788 10.1016/S0020-1383(09)70009-9

[19] Fujisawa R, Wada Y, Nodasaka Y, Kuboki Y. Acidic amino acid-rich sequences as binding sites of osteonectin to hydroxyapatite crystals. Biochim Biophys Acta. 1996;1292(1):53–60. 8547349 10.1016/0167-4838(95)00190-5

[20] Oldberg A, Franzen A, Heinegard D. The primary structure of a cell-binding bone sialoprotein. J Biol Chem. 1988;263(36):19430–2. 3198635

[21] Goldberg HA, Warner KJ, Li MC, Hunter GK. Binding of bone sialoprotein, osteopontin and synthetic polypeptides to hydroxyapatite. Connect Tissue Res. 2001;42(1):25–37. 11696986 10.3109/03008200109014246

[22] Culpepper BK, Phipps MC, Bonvallet PP, Bellis SL. Enhancement of peptide coupling to hydroxyapatite and implant osseointegration through collagen mimetic peptide modified with a polyglutamate domain. Biomaterials. 2010;31(36):9586–94. 10.1016/j.biomaterials.2010.08.020 21035181 PMC2991135

[23] Sawyer AA, Weeks DM, Kelpke SS, McCracken MS, Bellis SL. The effect of the addition of a polyglutamate motif to RGD on peptide tethering to hydroxyapatite and the promotion of mesenchymal stem cell adhesion. Biomaterials. 2005;26(34):7046–56. 10.1016/j.biomaterials.2005.05.006 15964067

[24] Sawyer AA, Hennessy KM, Bellis SL. The effect of adsorbed serum proteins, RGD and proteoglycan-binding peptides on the adhesion of mesenchymal stem cells to hydroxyapatite. Biomaterials. 2007;28(3):383–92. 10.1016/j.biomaterials.2006.08.031 16952395

[25] Hennessy KM, Pollot BE, Clem WC, Phipps MC, Sawyer AA, Culpepper BK, et al The effect of collagen I mimetic peptides on mesenchymal stem cell adhesion and differentiation, and on bone formation at hydroxyapatite surfaces. Biomaterials. 2009;30(10):1898–909. 10.1016/j.biomaterials.2008.12.053 19157536 PMC3679919

[26] Gilbert M, Shaw WJ, Long JR, Nelson K, Drobny GP, Giachelli CM, et al Chimeric peptides of statherin and osteopontin that bind hydroxyapatite and mediate cell adhesion. J Biol Chem. 2000;275(21):16213–8. 10.1074/jbc.M001773200 10748043

[27] Fujisawa R, Mizuno M, Nodasaka Y, Kuboki Y. Attachment of osteoblastic cells to hydroxyapatite crystals by a synthetic peptide (Glu7-Pro-Arg-Gly-Asp-Thr) containing two functional sequences of bone sialoprotein. Matrix Biol. 1997;16(1):21–8. 9181551 10.1016/s0945-053x(97)90113-x

[28] Itoh D, Yoneda S, Kuroda S, Kondo H, Umezawa A, Ohya K, et al Enhancement of osteogenesis on hydroxyapatite surface coated with synthetic peptide (EEEEEEEPRGDT) in vitro. J Biomed Mater Res. 2002;62(2):292–8. 10.1002/jbm.10338 12209950

[29] Murphy MB, Hartgerink JD, Goepferich A, Mikos AG. Synthesis and in vitro hydroxyapatite binding of peptides conjugated to calcium-binding moieties. Biomacromolecules. 2007;8(7):2237–43. 10.1021/bm070121s 17530891

[30] Pensa NW, Curry AS, Reddy MS, Bellis SL. The addition of a polyglutamate domain to the angiogenic QK peptide improves peptide coupling to bone graft materials leading to enhanced endothelial cell activation. PLoS One. 2019;14(3):e0213592 10.1371/journal.pone.0213592 30856221 PMC6411101

[31] Bain JL, Bonvallet PP, Abou-Arraj RV, Schupbach P, Reddy MS, Bellis SL. Enhancement of the regenerative potential of anorganic bovine bone graft utilizing a polyglutamate-modified BMP2 peptide with improved binding to calcium-containing materials. Tissue Eng Part A. 2015;21(17–18):2426–36. 10.1089/ten.TEA.2015.0160 26176902 PMC4555648

[32] Bain JL, Culpepper BK, Reddy MS, Bellis SL. Comparing variable-length polyglutamate domains to anchor an osteoinductive collagen-mimetic peptide to diverse bone grafting materials. Int J Oral Maxillofac Implants. 2014;29(6):1437–45. 10.11607/jomi.3759 25397807 PMC4504020

[33] Culpepper BK, Bonvallet PP, Reddy MS, Ponnazhagan S, Bellis SL. Polyglutamate directed coupling of bioactive peptides for the delivery of osteoinductive signals on allograft bone. Biomaterials. 2013;34(5):1506–13. 10.1016/j.biomaterials.2012.10.046 23182349 PMC3518561

[34] Culpepper BK, Webb WM, Bonvallet PP, Bellis SL. Tunable delivery of bioactive peptides from hydroxyapatite biomaterials and allograft bone using variable-length polyglutamate domains. J Biomed Mater Res A. 2014;102(4):1008–16. 10.1002/jbm.a.34766 23625466 PMC3808508

[35] Long S, Truong L, Bennett K, Phillips A, Wong-Staal F, Ma H. Expression, purification, and renaturation of bone morphogenetic protein-2 from Escherichia coli. Protein Expr Purif. 2006;46(2):374–8. 10.1016/j.pep.2005.09.025 16298141

[36] Kilpadi KL, Chang PL, Bellis SL. Hydroxylapatite binds more serum proteins, purified integrins, and osteoblast precursor cells than titanium or steel. J Biomed Mater Res. 2001;57(2):258–67. 11484189 10.1002/1097-4636(200111)57:2<258::aid-jbm1166>3.0.co;2-r

[37] von Einem S, Schwarz E, Rudolph R. A novel TWO-STEP renaturation procedure for efficient production of recombinant BMP-2. Protein Expr Purif. 2010;73(1):65–9. 10.1016/j.pep.2010.03.009 20302941

[38] Bragdon B, Moseychuk O, Saldanha S, King D, Julian J, Nohe A. Bone Morphogenetic Proteins: A critical review. Cell Signal. 2011;23(4):609–20. 10.1016/j.cellsig.2010.10.003 20959140

[39] Vallejo LF, Rinas U. Optimized procedure for renaturation of recombinant human bone morphogenetic protein-2 at high protein concentration. Biotechnol Bioeng. 2004;85(6):601–9. 10.1002/bit.10906 14966801

[40] Constam DB, Robertson EJ. Regulation of bone morphogenetic protein activity by pro domains and proprotein convertases. J Cell Biol. 1999;144(1):139–49. 10.1083/jcb.144.1.139 9885250 PMC2148113

[41] Ruppert R, Hoffmann E, Sebald W. Human bone morphogenetic protein 2 contains a heparin-binding site which modifies its biological activity. Eur J Biochem. 1996;237(1):295–302. 8620887 10.1111/j.1432-1033.1996.0295n.x

[42] Ohkawara B, Iemura S, ten Dijke P, Ueno N. Action range of BMP is defined by its N-terminal basic amino acid core. Curr Biol. 2002;12(3):205–9. 11839272 10.1016/s0960-9822(01)00684-4

[43] Zhang Y, Ma Y, Yang M, Min S, Yao J, Zhu L. Expression, purification, and refolding of a recombinant human bone morphogenetic protein 2 in vitro. Protein Expr Purif. 2011;75(2):155–60. 10.1016/j.pep.2010.07.014 20691269

[44] Billings PC, Yang E, Mundy C, Pacifici M. Domains with highest heparan sulfate-binding affinity reside at opposite ends in BMP2/4 versus BMP5/6/7: Implications for function. J Biol Chem. 2018;293(37):14371–83. 10.1074/jbc.RA118.003191 30082319 PMC6139562

[45] Rider CC, Mulloy B. Bone morphogenetic protein and growth differentiation factor cytokine families and their protein antagonists. Biochem J. 2010;429(1):1–12. 10.1042/BJ20100305 20545624

[46] Gandhi NS, Mancera RL. Prediction of heparin binding sites in bone morphogenetic proteins (BMPs). Biochim Biophys Acta. 2012;1824(12):1374–81. 10.1016/j.bbapap.2012.07.002 22824487

[47] Chenard KE, Teven CM, He TC, Reid RR. Bone morphogenetic proteins in craniofacial surgery: current techniques, clinical experiences, and the future of personalized stem cell therapy. J Biomed Biotechnol. 2012;2012:601549 10.1155/2012/601549 23226941 PMC3511855

