# Supplementary figures and images for: Addition of an oligoglutamate domain to bone morphogenic protein 2 confers binding to hydroxyapatite materials and induces osteoblastic signaling
Source: PLoS One. 2019 May 31;14(5):e0217766. doi: 10.1371/journal.pone.0217766 (PMC6544276; doi:10.1371/journal.pone.0217766)

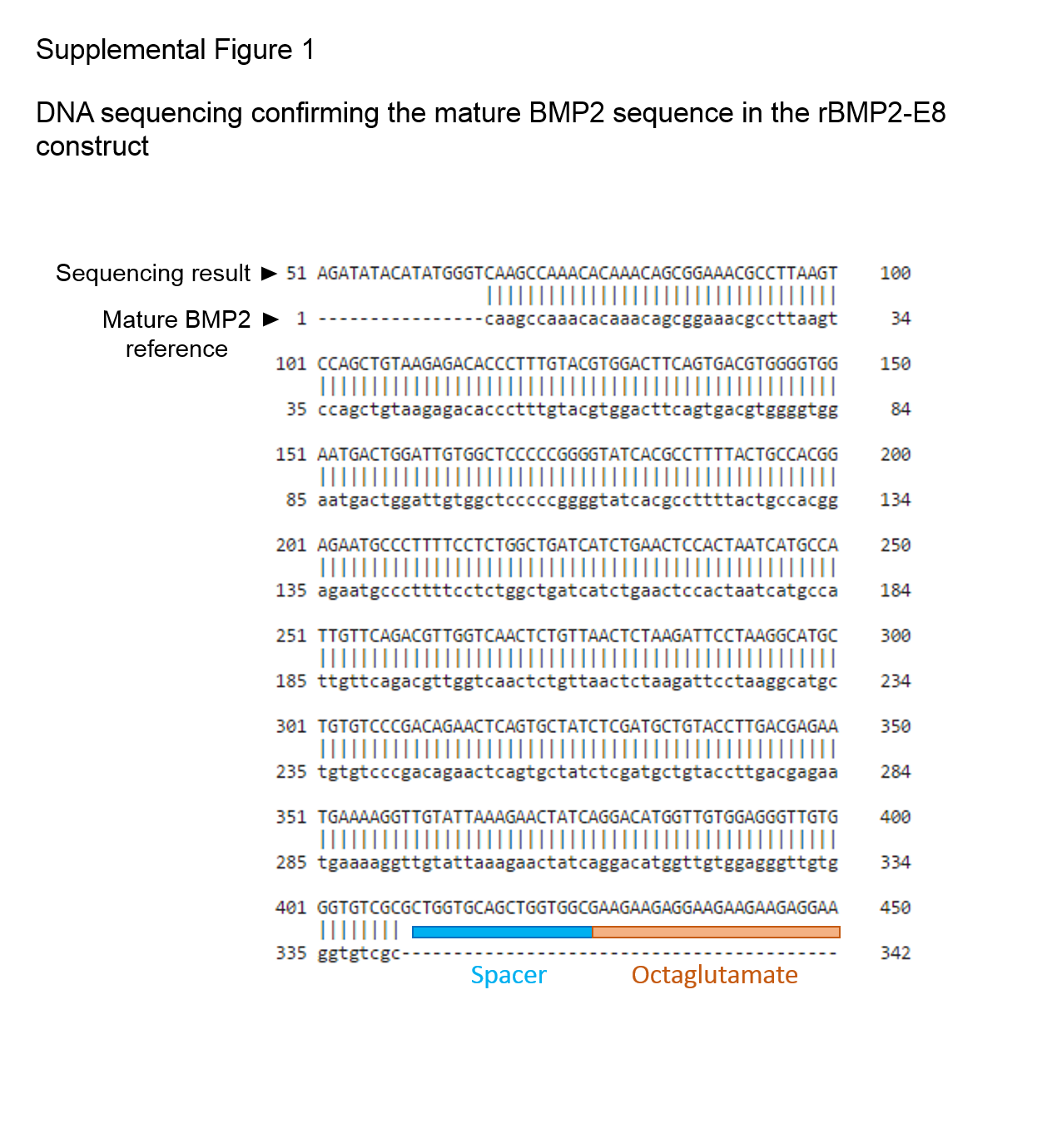

Supplement: S1 Fig — (TIF) [file pone.0217766.s001.tif]

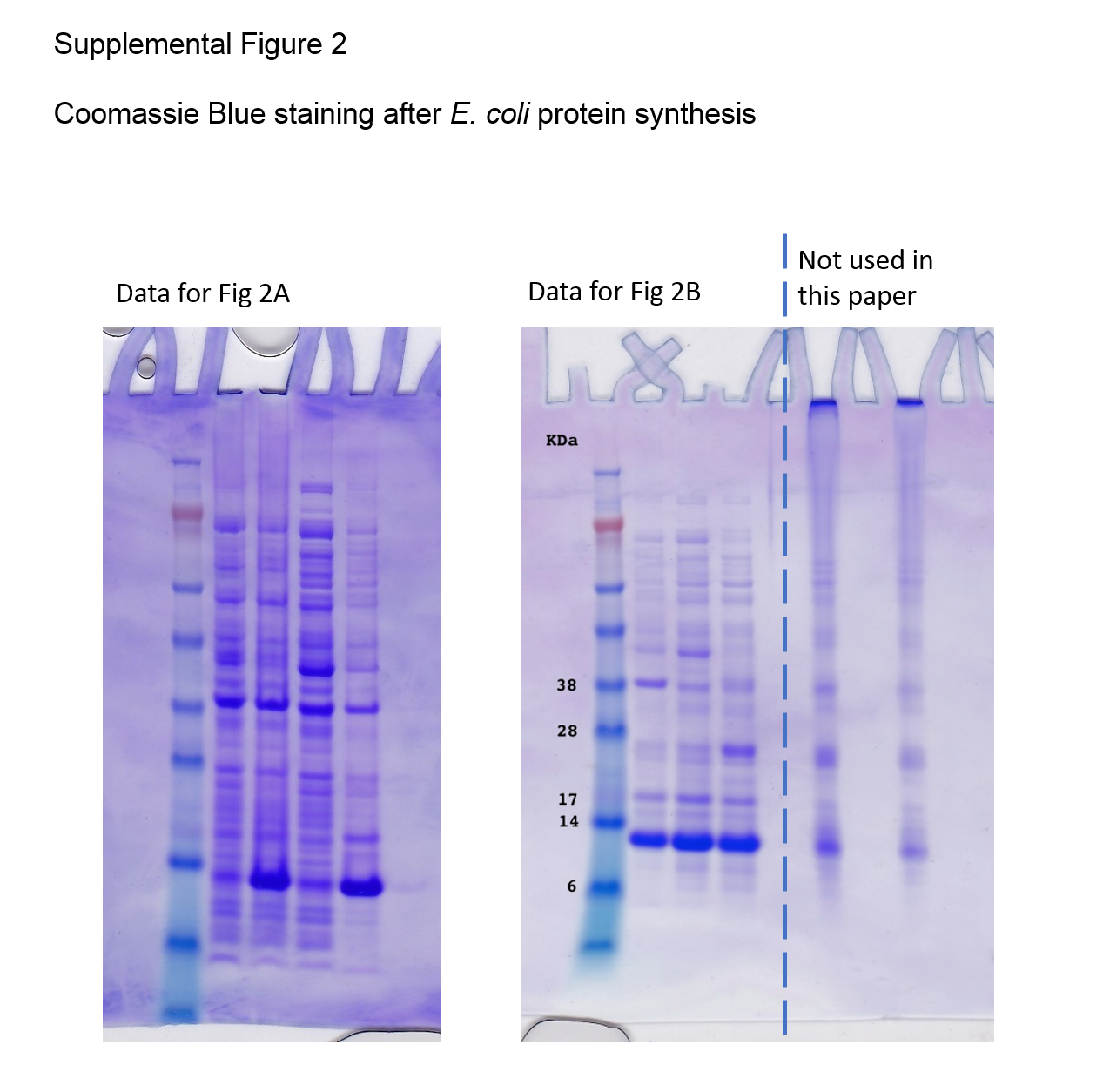

Supplement: S2 Fig — (TIF) [file pone.0217766.s002.tif]

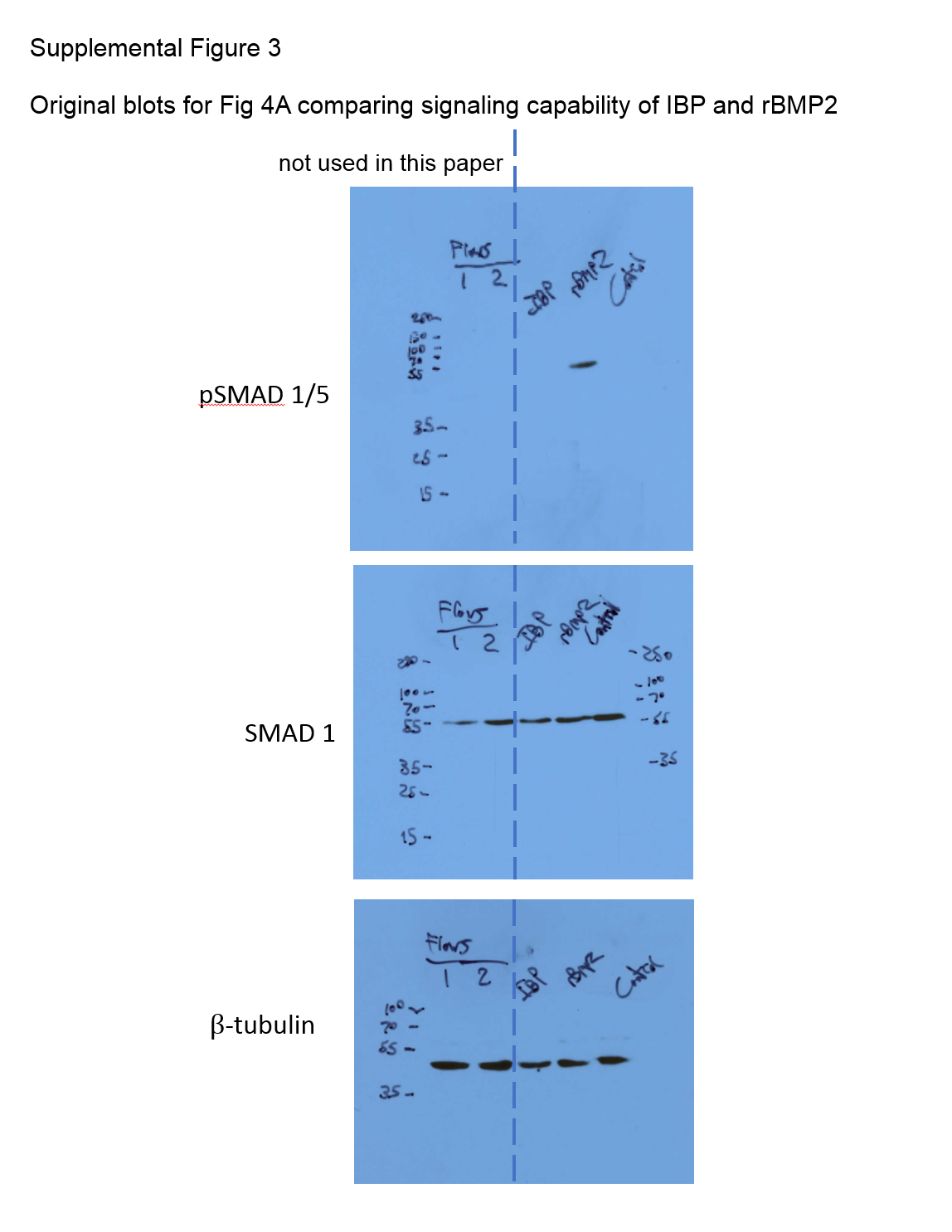

Supplement: S3 Fig — (TIF) [file pone.0217766.s003.tif]

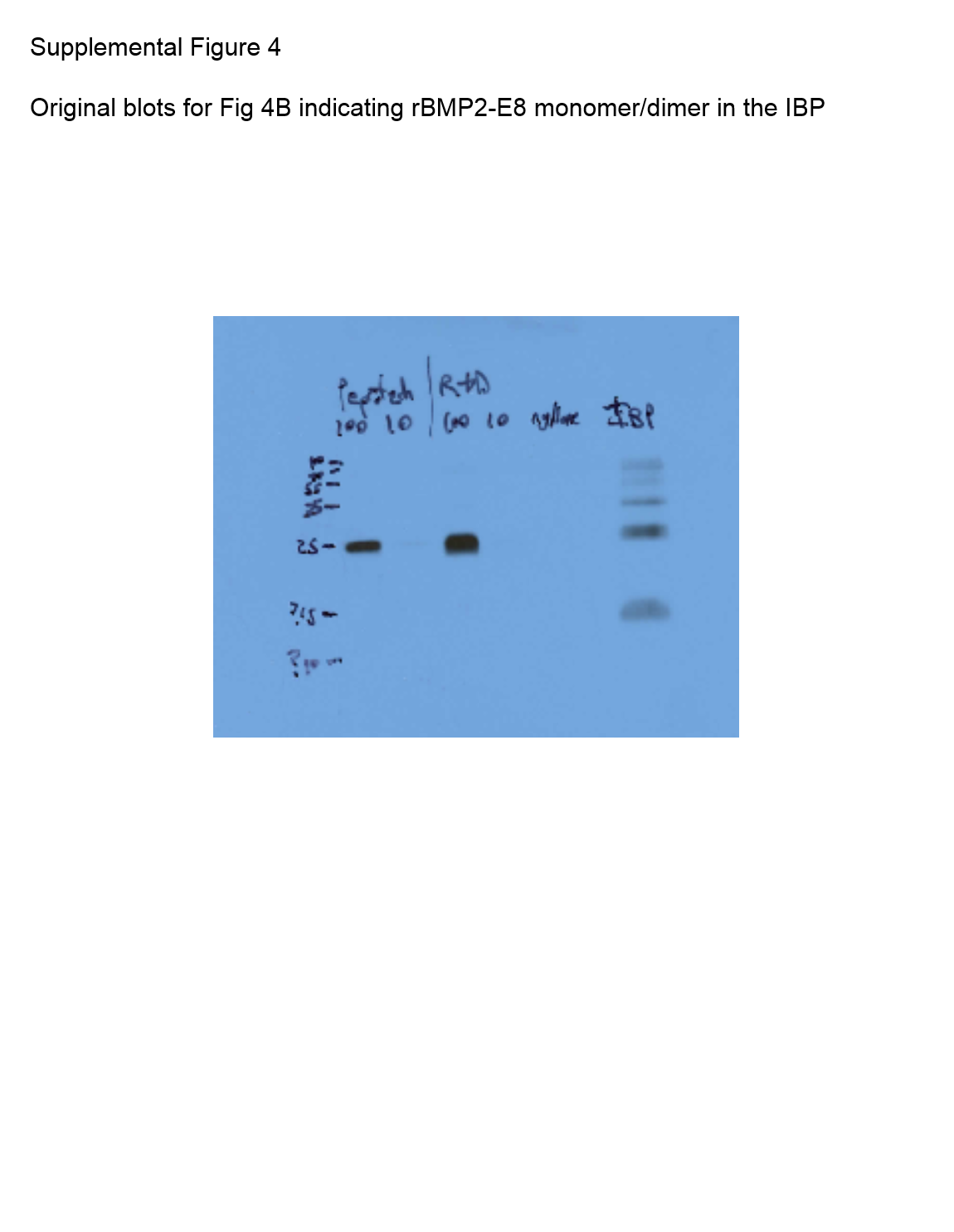

Supplement: S4 Fig — (TIF) [file pone.0217766.s004.tif]

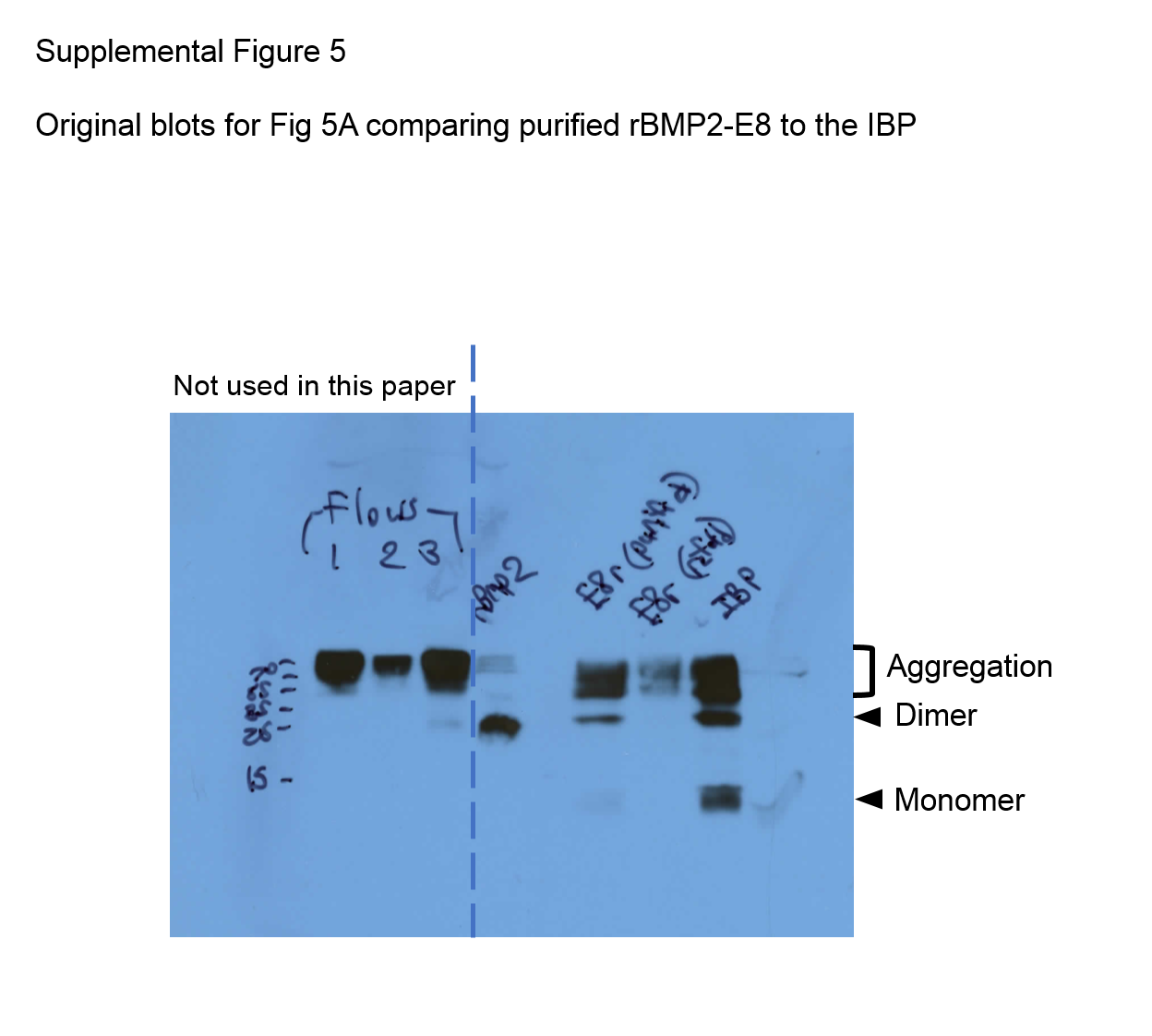

Supplement: S5 Fig — (TIF) [file pone.0217766.s005.tif]

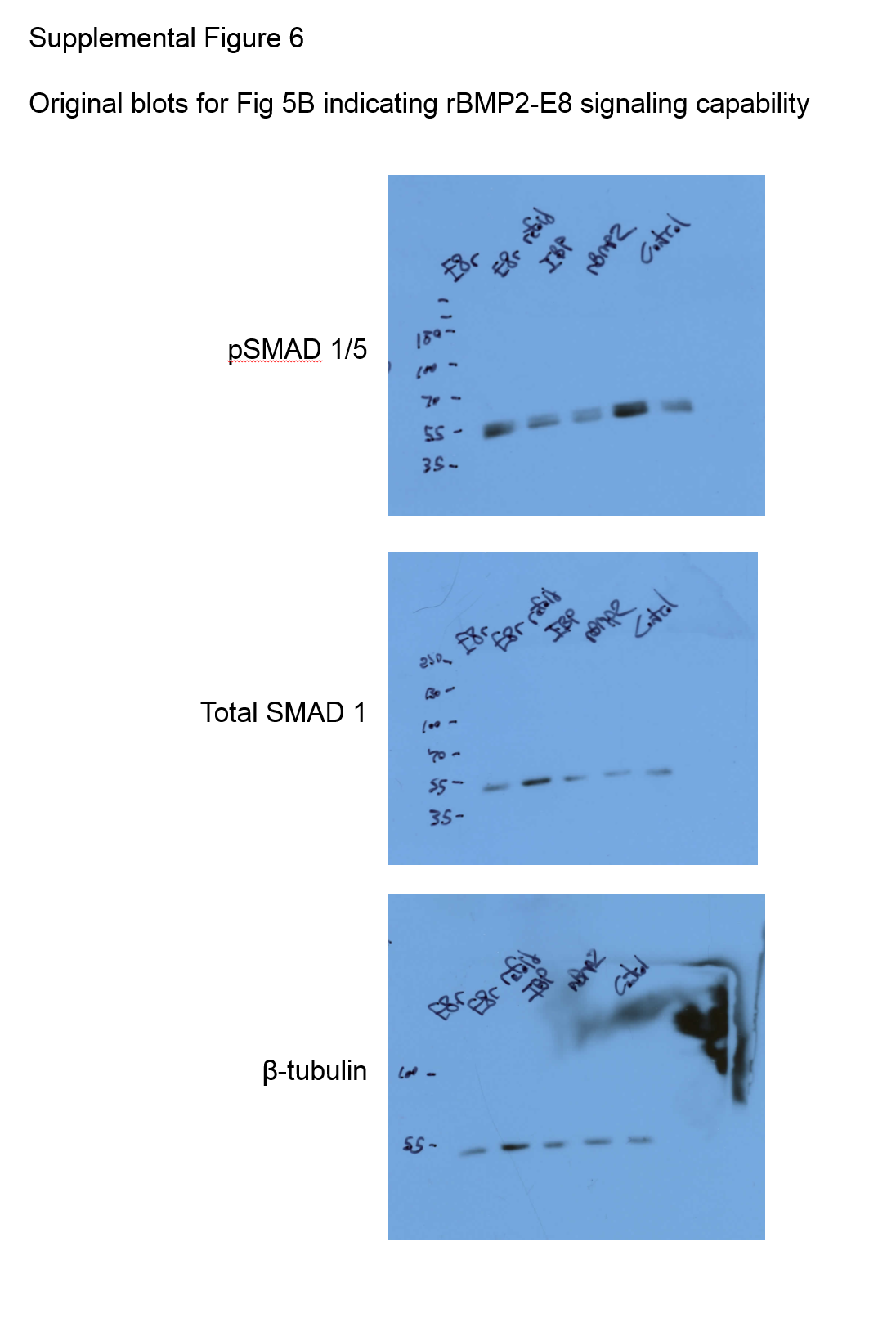

Supplement: S6 Fig — (TIF) [file pone.0217766.s006.tif]
